# Wie viel Digital Public Health steckt in Public-Health-Studiengängen? Eine systematische Modulhandbuchanalyse von Vollzeitstudiengängen an öffentlichen Hochschulen und Universitäten in Deutschland

**DOI:** 10.1007/s00103-024-03844-2

**Published:** 2024-03-04

**Authors:** Joanna Albrecht, Laura Maaß, Pinar Tokgöz, Robert Hrynyschyn, Kamil J. Wrona, Anna Lea Stark, Celina Dunsche, Florian Fischer, Annalena Schmidt, Henriette Schulz, Sarah Hidding, Christoph Dockweiler

**Affiliations:** 1 Universität Siegen, Lebenswissenschaftliche Fakultät, Department Digitale Gesundheitswissenschaften und Biomedizin, Professur für Digital Public Health, Siegen, Deutschland; 2 Leibniz WissenschaftsCampus Digital Public Health Bremen, Bremen, Deutschland; 3 Universität Bremen, Forschungszentrum Ungleichheit und Sozialpolitik (SOCIUM), Mary-Somerville-Straße 3, 28359 Bremen, Deutschland; 4 Charité-Universitätsmedizin Berlin, corporate member of Freie Universität Berlin and Humboldt-Universität zu Berlin, Institut für Gesundheits- und Pflegewissenschaft, Berlin, Deutschland; 5 Hochschule Bielefeld, Fachbereich Ingenieurwissenschaften und Mathematik, Bielefeld, Deutschland; 6 Hochschule Bielefeld, Fachbereich Gesundheit, Bielefeld, Deutschland; 7 Charité-Universitätsmedizin Berlin, corporate member of Freie Universität Berlin and Humboldt-Universität zu Berlin, Institut für Public Health, Berlin, Deutschland; 8 Hochschule Kempten, Bayerisches Zentrum Pflege Digital, Kempten, Deutschland; 9 Universität Bremen, Fachbereich Human- und Gesundheitswissenschaften, Bremen, Deutschland; 10 Deutsche Gesellschaft für Public Health e.V., Fachbereich Digital Public Health, Berlin, Deutschland

**Keywords:** Lehre, Digitalisierung, Deutsche Gesellschaft für Public Health, Gesundheitswissenschaften, Studiengang, Higher education teaching, Digitalization, German Public Health Association, Health sciences, Study course

## Abstract

**Hintergrund:**

Fachkräfte für den Bereich Digital Public Health (DiPH) sind für eine erfolgreiche digitale Transformation im Sozial- und Gesundheitswesen notwendig. Unklar ist jedoch, inwiefern im Public-Health-(PH-)Studium DiPH-bezogene Inhalte vermittelt werden.

**Methode:**

Mittels systematischer Modulhandbuchanalyse wurden DiPH-bezogene Inhalte von akkreditierten PH-orientierten Studiengängen öffentlicher Hochschulen und Universitäten in Deutschland analysiert. Über die Plattform „Hochschulkompass“ und Mitgliedsstudiengänge der Deutschen Gesellschaft für Public Health (DGPH) wurden 422 Studiengänge identifiziert. Eingeschlossene Modulhandbücher wurden inhaltsanalytisch mittels MAXQDA ausgewertet.

**Ergebnisse:**

Lediglich 10 Bachelor- und 6 Masterstudiengänge weisen einen inhaltlichen DiPH-Bezug auf. Sie sind in ihren Schwerpunkten heterogen und unterschiedlichen PH-Teilbereichen zuzuordnen („Methoden, Definition, Geschichte und Sozialmedizin“ = 5; „Gesundheitsmanagement“ = 5; „Digital Health“ = 3; „Versorgungsforschung“ = 2; „Gesundheitskommunikation“ = 1). Zwischen dem wissenschaftlich gängigen Verständnis von DiPH und den darauf bezogenen Inhalten in den Modulhandbüchern zeigen sich Unterschiede. Die identifizierten Inhalte fokussieren eher technische und geringfügiger sozial- und gesundheitswissenschaftliche Bereiche.

**Diskussion:**

Die heterogenen Studiengänge mit DiPH-Bezug ermöglichen akademischen PH-Fachkräften eine spezifische Profilierung. Um umfassende Kompetenzen im Bereich DiPH zu erlangen, bedarf es eines weiteren Ausbaus entsprechender Module, die für den jeweiligen Studiengang relevant sind. Die Ergebnisse könnten zur (Weiter‑)Entwicklung geeigneter Module sowie eines DiPH-Kerncurriculums dienen.

**Zusatzmaterial online:**

Zusätzliche Informationen sind in der Online-Version dieses Artikels (10.1007/s00103-024-03844-2) enthalten.

## Abstract (en)

**Background:**

Professionals, especially in the field of digital public health (DiPH), are crucial for a successful digital transformation in social and health care. However, it is still unclear to what extent academic professionals are taught DiPH-related content in their public health (PH) studies.

**Methods:**

This study used a systematic module handbook analysis to analyze accredited full-time PH-oriented degree programs at public colleges and universities in Germany for DiPH-related module content. Through the “Hochschulkompass” platform and the member programs of the German Public Health Association (DGPH), 422 programs were identified. Included module handbooks were evaluated by content analysis using MAXQDA.

**Results:**

Only 10 bachelor and 6 master programs contain DiPH. They are heterogeneous in their focus and belong to different subfields of public health (“methods, definition, history, and social medicine” = 5; “health management” = 5; “digital health” = 3; “health services research” = 2; “health communication” = 1). Differences were found between the common understanding of DiPH in academia and the content in the module handbooks. The content identified in the analysis focuses mainly on technical areas. Social and health science content is only marginally present.

**Discussion:**

The heterogeneous study programs with a connection to DiPH allow academic PH specialists to develop specific profiles. To achieve comprehensive competencies in DiPH, there is a need for further development of modules with relevance to the respective degree program. The results could be used for the (further) development of relevant modules and a core curriculum in DiPH.

## Einleitung

Digitale Lösungen bieten im Gesundheitswesen die Möglichkeit, Abläufe und Arbeitsweisen effizienter und effektiver zu gestalten [1]. Ebenso können Maßnahmen zur Gesundheitsförderung und Prävention kosteneffizienter und niederschwelliger durchgeführt sowie neue Versorgungsmöglichkeiten etabliert werden[2]. Nicht zuletzt offenbarte die COVID-19-Pandemie einige Defizite öffentlicher Gesundheitssysteme [3] und beschleunigte den Einsatz digitaler Technologien maßgeblich [4].

Die erfolgreiche Weiterentwicklung der Digitalisierung in Deutschland hängt vorrangig von einer angemessenen Qualifikation des Fachpersonals ab [4], denn in einer zunehmend digitalisierten Welt sind Fähigkeiten im Bereich der digitalen Gesundheit zu einem entscheidenden Faktor geworden [5]. Obwohl die Stärken digitaler Gesundheitstechnologien deutlich sind, können sie erst ihr volles Potenzial entfalten, wenn sichergestellt wird, dass die kommende Generation von Fachkräften im Bereich Public Health (PH) in der Lage ist, diese Technologien effektiv und verantwortungsbewusst einzusetzen [5, 6]. Daher ist es von großer Bedeutung, dass Bildungsprogramme diese Veränderungen widerspiegeln und sicherstellen, dass Absolvent*innen im Bereich PH über das erforderliche Fachwissen und die notwendigen Fähigkeiten verfügen, um künftigen Herausforderungen in der digitalen Gesundheitslandschaft gerecht zu werden [6, 7]. Eine mögliche Herangehensweise ist die Etablierung und regelmäßige Aktualisierung definierter Kriterien und Inhalte im PH-Studium, ähnlich wie bei der bundeseinheitlichen Approbationsordnung für Ärzt*innen [8, 9]. Eine Vereinheitlichung definierter Kriterien und Inhalte im PH-Studium könnte sicherstellen, dass PH-Fachkräfte kontinuierlich auf Herausforderungen der sich wandelnden Gesundheitslandschaft vorbereitet werden.

**Figure 1:**
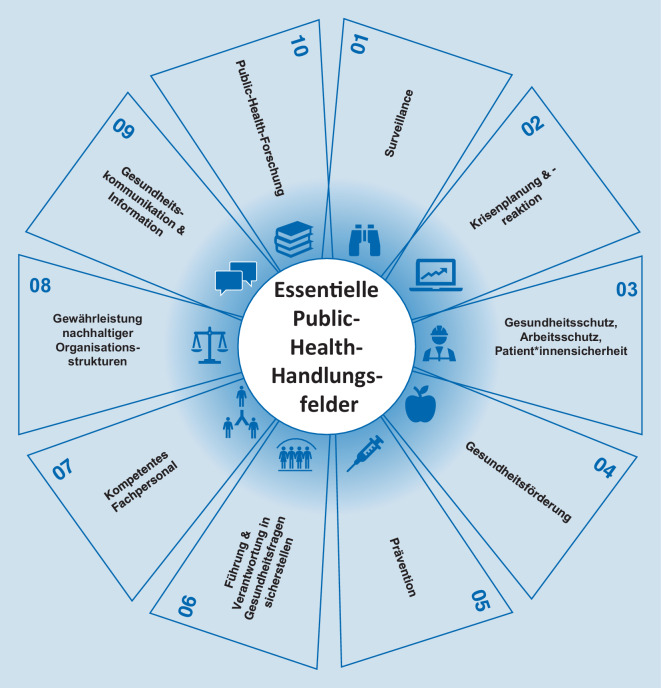

PH ist nach Acheson die „Wissenschaft und Praxis der Prävention von Krankheiten, der Verlängerung des Lebens und der Förderung, des Schutzes und der Verbesserung der Gesundheit durch die organisierten Bemühungen der Gesellschaft“ [10]. Mit der zunehmenden Digitalisierung in Aufgabenbereichen von PH stellt Digital Public Health (DiPH) keine neue Disziplin dar, sondern eher ein Hilfsmittel, um allgemeine PH-Ziele (vgl. *Essential Public Health Operations* – EPHO, Abb. 1) durch den Einsatz von Informations- und Kommunikationstechnologien (IKT) zu erreichen [11, 12]. Aufgrund der Vielfalt dieser Handlungsfelder reichen entsprechende DiPH-Interventionen von Systemdiensten wie elektronischen Patientenakten über den Einsatz von Webseiten, das digitale Gesundheitsmonitoring bis hin zu gesundheitsfördernden Apps [13]. DiPH kombiniert folglich technische Themengebiete wie Ingenieurs- und Computerwissenschaften mit den klassischen PH-Feldern aus Sozial‑, Human- und Umweltwissenschaften [14]. Der Fokus liegt bei DiPH wie auch bei PH auf der Erhaltung und Förderung der Gesundheit von Bevölkerungsgruppen [5, 15, 16].

Drei Scoping-Reviews zu erforderlichen digitalen Kompetenzen von PH-Fachkräften [19–21] ergaben folgende fachlichen Anforderungen:

1. sichere Beherrschung von IT-Anwendungen zur Datenanalyse und -aufbereitung,
2. ausreichende digitale (Gesundheits‑)Kompetenzen (einschließlich technischer Aspekte von IT-Anwendungen wie Softwareanwendungen, Tests und angewandter Informatik),
3. Sensibilisierung für kulturelle Einflüsse im Einsatz von IT in der Patientenversorgung,
4. Kenntnisse zu ethischen und rechtlichen Richtlinien im Umgang mit digitalen Gesundheitsdaten,
5. sicherer Umgang mit elektronischen Gesundheitsdatensätzen und Wissensmanagement,
6. Qualitätsmanagement und Sicherheit von Gesundheitsdiensten,
7. Kenntnisse über integrierte Gesundheits-IT-Anwendungen,
8. Nutzungskompetenzen für IT-Anwendungen zur Unterstützung von Forschung, Interventionsentwicklung und -implementierung sowie für deren Verwendung als Informationsmedium für PH-Strategien,
9. Förderung der Nutzung von IT-Anwendungen in der Gesundheitsförderung und Patientenversorgung sowie Befähigung der Patient*innen zu einer selbstbestimmten Nutzung.

Die Breite an Fachkompetenzen erfordert eine kritische Analyse, ob sich diese auch in den Ausbildungsinhalten der PH-Fachkräfte widerspiegelt. Das Primärziel dieser Arbeit war es daher, zu identifizieren, ob öffentliche Hochschulen in Deutschland ihren PH-Studierenden derzeit gesonderte DiPH-Module anbieten, um sie so auf die Berufspraxis vorzubereiten. Das sekundäre Ziel war herauszufinden, welche Inhalte die entsprechenden Module vermitteln und ob sich diese von den identifizierten Kompetenzen von PH-Praktiker*innen und Forscher*innen unterscheiden.

## Methoden

Es wurde eine qualitativ deskriptive Modulhandbuchanalyse (MHBA) durchgeführt. Dabei wurden die Modulhandbücher (MHB) akkreditierter PH-orientierter Studiengänge an öffentlichen Hochschulen und Universitäten in Deutschland hinsichtlich ihrer DiPH-bezogenen Inhalte analysiert.

### Recherche und Einschlusskriterien

Analysiert wurden Studiengänge von Institutionen, die zum 20.11.2022 als Mitglieder der Deutschen Gesellschaft für Public Health e. V. (DGPH; [22]) gelistet waren oder über die Plattform „Hochschulkompass“ mittels der Suchbegriffe „Public Health“ und „Gesundheitswissenschaften“ identifiziert werden konnten [23]. Es wurden öffentlich-rechtliche Universitäten, (Fach‑)Hochschulen und Hochschulen für Angewandte Wissenschaft (HAW) in Deutschland eingeschlossen. Die Auswahl beschränkte sich auf Vollzeitstudiengänge. Über die DGPH-Mitgliedersuche konnten 35 Studiengänge (9 Bachelor- und 26 Masterstudiengänge) identifiziert werden. Die Suche im „Hochschulkompass“ ergab für „Public Health“ 87 Ergebnisse (30 Bachelor- und 57 Masterstudiengänge) und für „Gesundheitswissenschaften“ 498 Treffer (203 Bachelor-, 221 Masterstudiengänge und 74 Studiengänge auf Staatsexamen), die in den Auswahlprozess einbezogen wurden.

### Screening der Modulhandbücher

Zunächst wurde festgestellt, ob es sich bei den vorliegenden Studiengängen inhaltlich um PH-orientierte Studiengänge handelte. Hierfür wurden die PH-Definition der DGPH, der PH-Fachqualifizierungsrahmen und die PH-Kernkompetenzen laut der „Association of Schools of Public Health in the European Region“ (ASPHER; [24–26]) herangezogen. Diese ergaben 7 Kernthemen für einen PH-Studiengang:

1. Public Health/Sozialmedizin,
2. Public-Health-Forschungsmethoden/Sozial- und Versorgungsforschung,
3. Epidemiologie,
4. Gesundheitsförderung/Prävention/Gesundheitserziehung,
5. Gesundheits- und Krankheitsdeterminanten/soziale Ungleichheit,
6. Gesundheitsökonomie und -management,
7. Gesundheitspolitik und -system.

Um sicherzustellen, dass diese Themen in ausreichender Tiefe gelehrt wurden, mussten die dazugehörigen Module im Studiengang mit mindestens 5 Credit Points (CP), Leistungspunkten (LP) oder European Credit Transfer System (ECTS) gewichtet sein (nachfolgend wird zur Angabe der Gewichtung das Kürzel CP verwendet). Dies entsprach einem Umfang von 125–150 h pro Semester (ca. 9–11 Wochenstunden). Wies ein Modul Inhalte mehrerer Kerndisziplinen auf, wurden die CP allen angesprochenen Kerndisziplinen zugeordnet. Es wurde nicht berücksichtigt, ob einzelne Module nur innerhalb eines bestimmten Schwerpunktes oder als Wahlmodul angeboten wurden. Alle im MHB aufgeführten und belegbaren Module wurden in die Analyse einbezogen. Diese Vorgaben wurden anhand der 39 DGPH-Mitgliedsstudiengänge auf ihre Machbarkeit getestet. Jedoch erfüllten nur 14 Studiengänge diese Vorgaben. Die Anforderung, 7 Kernthemen mit je 5 CP zu erfüllen, wurde daher auf die Anforderung, 5 der 7 Kernthemen mit je 5 CP zu erfüllen, reduziert, woraufhin 31 von 39 Studiengängen die Vorgabe von mindestens 5 CP in 5 der oben benannten Kernthemen erfüllten.

Zusätzlich zu den verpflichtenden Kernthemen wurden 4 Querschnittsthemen nach erster Sichtung von Modulhandbüchern deduktiv definiert, welche für PH-Studiengänge fakultativ sein können: Gesundheitskommunikation, Ethik, Global Health und Digital Health. Anschließend wurde erhoben, ob und, wenn ja, wie viele und in welchem Umfang Module zu DiPH angeboten wurden. Die Lerninhalte der Module wurden für eine qualitative Datenanalyse extrahiert.

### Datenanalyse

Die Auswertung der eingeschlossenen Modulhandbücher erfolgte inhaltsanalytisch induktiv und deduktiv mittels MAXQDA (Version 22.4.1; VERBI – Software. Consult. Sozialforschung. GmbH, Berlin, Deutschland). Die deduktive Auswertung basierte auf dem oben dargelegten Verständnis von DiPH sowie den Screeningkriterien, wobei die definierten Teilbereiche sowie Querschnittsbereiche von PH als deduktive Kategorien fungierten. Bei der induktiven Auswertung wurden die Teilbereiche sowie Querschnittsbereiche von PH der DiPH-bezogenen Module um Unterkategorien ergänzt, die den Bereich inhaltlich differenzierter beschreiben (Tab. Z1 im Onlinematerial).

## Ergebnisse

**Figure 2:**
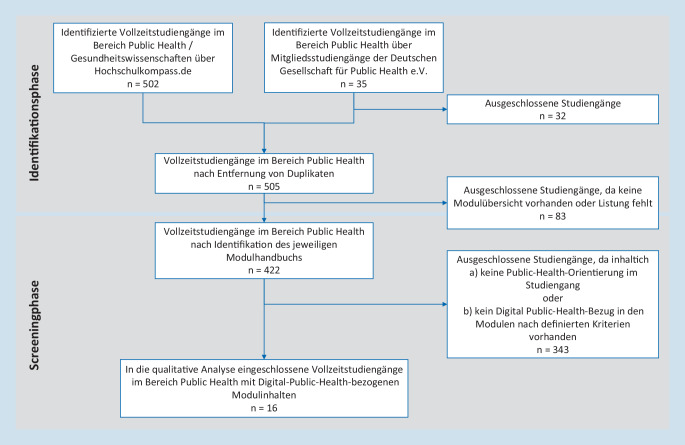

Mit Entfernung der Duplikate wurden die 620 recherchierten Vollzeitstudiengänge im Bereich PH auf 505 reduziert (Abb. 2). Bei 83 der identifizierten Studiengänge konnte keine Modulübersicht gefunden werden (z. B. über die Website, das MHB oder die Prüfungsordnung). 80 der 83 Studiengänge wurden aufgrund der Bezeichnung (z. B. (Zahn‑/Veterinär‑)Medizin, Hebammenkunde) als nicht-PH-relevant eingestuft und ausgeschlossen. Für die 3 verbleibenden Studiengänge ohne öffentlich einsehbare MHB wurden die Studienkoordinationen/-beratungen angefragt. Zwei MHB wurden zur Verfügung gestellt und nach Sichtung ausgeschlossen. Die weiteren 422 Studiengänge wurden in das Screening eingeschlossen. Nach Prüfung der Anforderung auf je 5 CP in 5 der 7 Kernthemen, wurden 79 Studiengänge als PH-orientiert eingestuft. Nach weiterer Prüfung auf DiPH-bezogene Inhalte wurden insgesamt 16 PH-orientierte Studiengänge in die qualitative Analyse eingeschlossen.

16 PH-orientierte Studiengänge an Universitäten und (Fach‑)Hochschulen in Deutschland wiesen in einem oder mehreren (Wahl‑)Pflichtmodulen einen inhaltlichen DiPH-Bezug auf (Tab. Z2 im Onlinematerial). Je 2 Studiengänge kamen aus Bochum, Fulda, Hamburg und Siegen und je ein Studiengang aus Aalen, Berlin, Bielefeld, Dresden, Furtwangen, Gießen, Kempten und Krefeld. Die Studiengänge waren primär an Hochschulen angesiedelt (*n* = 12), seltener an Universitäten (*n* = 4). Der Umfang der gesamten CP für DiPH-bezogene Module variierte zwischen 5 und 118 CP.

### PH-orientierte Studiengänge mit DiPH-Bezug – deskriptive Darlegung

In Tab. 1 wird die Anzahl der DiPH-bezogenen (Wahl‑)Pflichtmodule nach themenbezogenen Schwerpunkten der 16 analysierten PH-orientierten Studiengänge gruppiert. Der themenbezogene Schwerpunkt entspricht den Modulen zum Kernthema bzw. Querschnittsbereich mit der höchsten Anzahl der CP. Die themenbezogene Zuordnung der eingeschlossenen PH-Studiengänge umfasste folgende PH-Teilbereiche:

- *Management* (Gesundheitsmanagement [34]; Management und Versorgung im Gesundheitswesen [35]; Management in der Gesundheitsversorgung [36]; Interprofessionelles Management in der Gesundheitsversorgung [37] und Gesundheitswirtschaft [38]);
- *Gesundheitswissenschaften *(Gesundheitswissenschaften/PH [27]; PH [28]; Gesundheitswissenschaften [29]; Health Sciences [30] und Angewandte Gesundheitswissenschaften [31]) sowie
- *Versorgung* (Pflege [32] und Health Care [33]).

**Table 1:** 

Schwerpunkt des PH-orientierten Studiengangs	Studiengänge Modulhandbücher	Anzahl DiPH-bezogene Wahlpflicht-Module pro Studiengang (Min.–Max.)	Anzahl Credit Points (Min.–Max.)	Anzahl DiPH-bezogene Pflichtmodule pro Studiengang (Min.–Max.)	Anzahl Credit Points (Min.–Max.)
*PH-Kernthemen*					
Methoden, Definitionen und Geschichte von PH und Sozialmedizin	[27–31]	1–7	5–30	1–3	5–6
Versorgungsforschung und Forschungsmethoden in PH und der empirischen Sozialforschung	[32, 33]	–	–	5	5–8
Epidemiologie	Keine PH-orientierten Studiengänge mit dem Schwerpunkt Epidemiologie				
Gesundheitsförderung, Prävention und Gesundheitserziehung	Keine PH-orientierten Studiengänge mit dem Schwerpunkt Gesundheitsförderung, Prävention und Gesundheitserziehung				
Gesundheitsökonomie und Gesundheitsmanagement	[34–38]	1–3	5	1–3	5
Soziale, ökonomische, politische und umweltbedingte Determinanten von Gesundheit, Krankheit und sozialer Ungleichheit	Keine PH-orientierten Studiengänge mit dem Schwerpunkt soziale, ökonomische, politische und umweltbedingte Determinanten von Gesundheit, Krankheit und sozialer Ungleichheit				
Gesundheitspolitik und Gesundheitssystem	Keine PH-orientierten Studiengänge mit dem Schwerpunkt Gesundheitspolitik und Gesundheitssystem				
*(Transdisziplinäre) Querschnittsbereiche*					
Gesundheitskommunikation	[39]	2	5–20	2	5–10
Global Health	Keine PH-orientierten Studiengänge mit dem Schwerpunkt Global Health				
Ethik	Keine PH-orientierten Studiengänge mit dem Schwerpunkt Ethik				
Digital Health bzw. DiPH	[40–42]	9–14	6–12	4–8	3–7

*CP* Credit Points, *DiPH* Digital Public Health, *PH* Public Health

Darüber hinaus umschließen sie die Querschnittsbereiche:

d) *Gesundheitskommunikation* (Health Communication [39]) sowie
e) *Digital Health* (Gesundheitsdaten und Digitalisierung [40]; Digital Biomedical and Health Sciences (Vertiefung DiPH; [41]) und DiPH [42]).

Mit Blick auf die DiPH-Bezüge in den PH-orientierten Studiengängen zeigte sich, dass die höchste Anzahl von DiPH-bezogenen Modulen in den beiden Bachelorstudiengängen und einem Masterstudiengang mit Schwerpunkt DiPH vorkam [40–42]. Die 4 Bachelorstudiengänge und ein Masterstudiengang mit Schwerpunkt Management wiesen in je 1–3 Modulen einen DiPH-Bezug auf [34–38]. Weitere DiPH-Bezüge wurden in je 1–2 Wahlpflichtmodulen sowie in 1–3 Pflichtmodulen in den 4 Studiengängen mit dem Schwerpunkt Gesundheitswissenschaften verortet und dem Bereich Methoden, Definitionen und Geschichte von PH und Sozialmedizin zugeordnet [27–30]. Ferner wiesen die 2 Studiengänge mit Schwerpunkt Versorgungsforschung und Forschungsmethoden in PH und der empirischen Sozialforschung DiPH-Bezüge auf [32, 33]. Zuletzt wies ein Studiengang mit Schwerpunkt Gesundheitskommunikation DiPH-Bezüge mit je 2 Wahlpflicht- sowie Pflichtmodulen auf [39].

### Lehr- und Lerninhalte der DiPH-Module in PH-orientierten Studiengängen

**Figure 3:**
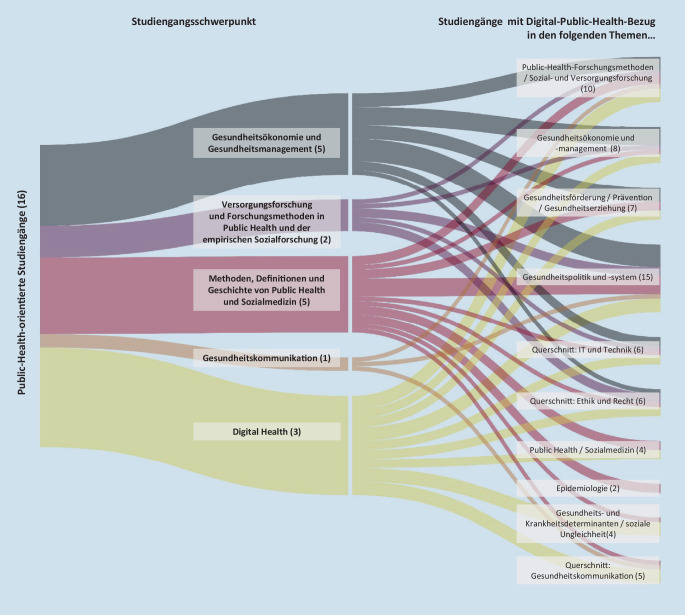

Die Darlegung der Lehr- und Lerninhalte der DiPH-bezogenen Module erfolgt entlang des entwickelten Kategoriensystems (Tab. Z1 im Onlinematerial). Die analysierten Module zeigten durch häufig breitgefächerte Inhalte Bezüge zu mehreren Kategorien auf (Abb. 3).

**Figure 4:**
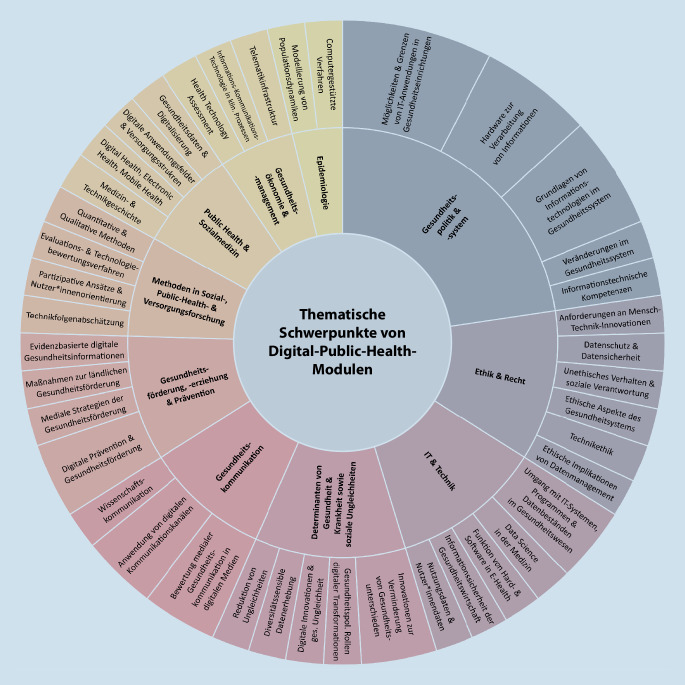

Die folgende Analyse bezieht sich nur auf die Themengebiete der DiPH-bezogenen Module, welche von mindestens 5 Studiengängen gelehrt wurden. Eine Gesamtübersicht der DiPH-bezogenen Module und ihrer Subthemen gibt Abb. 4.

#### Gesundheitspolitik und -system.

Wie Abb. 3 verdeutlicht, fokussierten die meisten Studiengänge mit DiPH-Bezug den Bereich der Gesundheitspolitik und Gesundheitssysteme [31–42]. Konkret wurde die zentrale Terminologie (z. B. Electronic Health (E-Health), Gesundheitstelematik und Telemedizin) aufgegriffen und auf verschiedene Aspekte von DiPH angewendet. Einzelne Studiengänge lehrten z. B. Grundlagen von IKT im Gesundheitssystem, wobei der Einsatz von Hardware zur Verarbeitung von Informationen, die kritische Reflexion von Möglichkeiten und Grenzen von IT-Anwendungen in Gesundheitseinrichtungen und -settings thematisiert wurden [34, 37, 40]. Bezüglich der Veränderungen in Gesundheitssystemen und -versorgung wurden Zusammenhänge zwischen Digitalisierungsprozessen sowie Veränderungspotenzialen, verschiedenen Anwendungsgebieten und Einsatzmöglichkeiten digitaler Technologien gelehrt [31–42]. Teils wurden diese mit technologiebezogenen Grundlagen zur Vermittlung informationstechnischer Kompetenzen aus der Systemperspektive verknüpft [28, 31, 34, 35, 37, 39–42].

#### Public-Health-Forschungsmethoden/Sozial- und Versorgungsforschung.

Der zweitgrößte inhaltliche DiPH-Block, die Anwendung von Methoden der Sozial‑, Public-Health- und Versorgungsforschung, wurde von 10 Studiengängen aufgegriffen [28–30, 32, 34, 36–42] und gliederte sich in 4 Themengebiete. Quantitative und qualitative Forschungsmethoden wurden in einigen Studiengängen praktisch auf DiPH-Themen, wie den Umgang mit Big Data [29], Projektmanagement [39] oder das Analysieren von Zusammenhängen im Sozialraum über Gesundheitsdaten, angewandt. Zudem befasste sich ein Studiengang mit der Vermittlung von Methoden im diversitätssensiblen Gesundheitsdatenmanagement, also unter Berücksichtigung der Vielfalt von Datennutzenden und -ersteller*innen [40]. Ebenso bot ein Studiengang Module zu Evaluations- und Technologiebewertungsverfahren an [34]. Thematisiert wurden auch Inhalte zur Konzeption von Webseiten mit Gesundheitsbezug [39], zu digitalen Diensten in der gesundheitlichen Versorgung und des Datenmanagements [40], Mensch-Technik-Innovationen [37], Beratungskonzepten zu Digitalisierung im Gesundheitswesen [29] oder Entwicklungsansätzen entlang des „Technological Hype Cycle“ (Methodik zur Ermittlung der Dynamik von Erwartungen in Innovationsprozessen; [31]). Im Sinne der Partizipation boten einige Studiengänge Module zur Analyse, Berücksichtigung und Durchführung von nutzer*innenorientierten Gestaltungs- und Entscheidungsprozessen an [31, 38, 40, 42]. Inhaltlich beschäftigten sich diese mit der Adoptions- und Akzeptanzforschung [41, 42], der Aufbereitung digitaler Informationen zur Zielgruppenanalyse [31, 39] oder der Entwicklung und Gestaltung von Anwendungsmöglichkeiten der Gesundheitsdaten [31, 40]. Die Studiengänge beschäftigten sich auch mit der aktiven Einbindung von Patient*innen in deren Behandlungen [32, 38] oder digitalen Interventionen in der Gesundheitsförderung und Prävention [42]. Den letzten DiPH-Schwerpunkt innerhalb der Sozialforschung stellte schließlich die Technikfolgenabschätzung dar, in der es um eine kritisch reflektierte Beleuchtung der Chancen, Folgen und Risiken von Anwendungsbereichen der Digitalisierung im Gesundheitswesen sowie die Identifikation (un)beabsichtigter gesellschaftlicher Folgen ging [29–31, 34, 40, 41]. Hier zeigten sich Schnittmengen mit den Querschnittsthemen *Ethik und Recht* sowie *IT und Technik*, da technische Grundlagen und Datenschutz gelehrt wurden, um Folgerisiken abschätzen zu können [29, 31, 41].

#### Gesundheitsökonomie und -management.

Die meisten DiPH-Module der 8 Studiengänge [30, 33–36, 38, 42] mit Bezug zu Gesundheitsökonomie und -management bezogen sich auf die gesundheitsökonomische Evaluation und Finanzierung von digitalen Leistungen. Dies beinhaltete sowohl Vergütungsmodelle (auf der Systemebene über die Telematikinfrastruktur und für konkrete E‑Health-Leistungen; [34]) als auch Grundlagen des Health Technology Assessment (HTA) am Beispiel digitaler Gesundheitsanwendungen für die Gesundheitsversorgung und Pflege. Hierbei wurden HTA-Berichte, Modellierungen der Auswirkungen auf Budget und Kostenwirksamkeit, wirtschaftliche Bewertungen von Primärstudien in Theorie und Praxis sowie die Einbindung von HTA-Berichten in verschiedenen Entscheidungskontexten vermittelt [30, 33, 34, 36, 38, 42]. Darüber hinaus wurde in einem Modul die Bedeutung von IKT für klinische Prozesse und den Gesundheitsmarkt betrachtet [35].

#### Gesundheitsförderung/Prävention/Gesundheitserziehung.

5 Studiengänge boten DiPH-Module im Bereich der Gesundheitserziehung, -förderung oder Prävention an [28, 31, 36, 41, 42]. Teilweise beschäftigten sich diese mit der qualitätsgesicherten und evidenzbasierten Gestaltung von gesundheitsbezogenen Informationen für Laien und PH-Fachkräfte in printbasierter oder digitaler Form [36]. Ferner wurden digitale Innovationen in der Gesundheitsförderung und Prävention eruiert, wobei die Einhaltung von Qualitätskriterien sowie die Anwendung im Kontext eines Rahmenmodells zur Interventionsentwicklung, dem „Public Health Action Cycle“, eingebettet wurden [41, 42]. Ebenso gab es Inhalte zu Bedarfen und Maßnahmen zur Gesundheitsförderung in ländlichen Gebieten [31]. Ein Modul zur ethischen Beurteilung medialer Strategien der Gesundheitsförderung wies Überschneidungen mit den Querschnittsthemen *Gesundheitskommunikation *sowie *Ethik und Recht* auf [28].

#### Querschnittsthemen.

Im Bereich *IT und Technik* befassten sich 6 Studiengänge [31, 33, 34, 38, 40, 41] mit dem Umgang mit IT-Systemen, Programmen und Datenbeständen, aber auch mit deren Anwendung im Gesundheitswesen bzw. in der -wirtschaft [31, 34, 40]. Ebenso wurden unterschiedliche Nutzungsdaten gelehrt [40]. Ein anderer Studiengang bot Module zum IT-Management in der Gesundheitswirtschaft mit einem Schwerpunkt auf Informationssicherheit in der Gesundheitswirtschaft und -versorgung [38]. Ebenso gab es Lehrinhalte zu Hard- und Softwareobjekten hinsichtlich ihrer Fertigungsverfahren (z. B. für smarte Technologien wie Smartwatches oder Sensoren) und Programmarchitektur [33]. Ein sechster Studiengang beleuchtete mithilfe der Datenwissenschaften (Data Science) bestehende sowie potenzielle Risiken und Herausforderungen wie auch aktuelle Trends und Entwicklungen in der Medizin [41].

Das Querschnittsthema *Ethik und Recht *fand sich in 7 Studiengängen wieder [30–33, 37, 40, 41]. Alle reflektierten ethische Implikationen im Datenmanagement sowie den Umgang mit Daten und Technik im Allgemeinen. Ein Studiengang ergänzte zentrale Aspekte der Technikethik und ethische Aspekte im Kontext individueller Gesundheit bzw. des Gesundheitssystems [40]. Im rechtlichen Kontext wurden insbesondere Aspekte des Datenschutzes und der Datensicherheit gelehrt [30, 32, 33, 40]. Zudem wurden ethische und rechtliche Anforderungen von Mensch-Technik-Innovationen sowie Tele-Health-Anwendungen in der Praxis thematisiert [33, 37, 41].

In der *Gesundheitskommunikation *lag der Schwerpunkt der 6 Studiengänge [28, 30, 39–42] auf den Theorien, Methoden, Prinzipien, Herausforderungen und der Anwendung von digitalen Kommunikationskanälen [28, 30, 39–42]. Vertiefend wurde vermittelt, wie die Kommunikationswissenschaften dabei helfen können, die digitale Gesundheitskommunikation zu analysieren und zu bewerten, wo ihre Grenzen liegen [28, 30, 40–42]. Darüber hinaus wurden Grundlagen der Wissenschaftskommunikation vermittelt [30, 42].

## Diskussion

In Deutschland wurden mit Stand: 11/2022 an insgesamt 12 Universitäts- und (Fach‑)Hochschulstandorten 16 PH-orientierte Studiengänge mit DiPH-bezogenen Modulen angeboten. Im Vergleich zu der Gesamtanzahl der als PH-orientiert identifizierten 79 Studiengänge ist dies ein verhältnismäßig geringer Anteil. Ferner sind die DiPH-bezogenen Module eher als Pflichtmodule und weniger als Wahlpflichtmodule konzipiert. Die Inhalte von DiPH-relevanten Modulen decken sich in Bezug auf Terminologie, Rahmenkonzepte, Modelle sowie Forschungsmethoden mit den zu Beginn dieser Arbeit identifizierten Zielen und Praxisanforderungen von PH und DiPH. Die Untersuchung dieser Module zeigt eine hohe Heterogenität, bedingt durch die spezifischen Ausrichtungen der verschiedenen Studienstandorte.

Wenn auch in einzelner Betrachtung der Modulhandbücher überwiegend einzelne DiPH-Inhalte fokussiert werden, weisen die aus den Modulhandbüchern ersichtlichen Inhalte insgesamt darauf hin, dass die erforderlichen Kompetenzen von PH-Fachkräften in Bezug auf die Digitalisierung [19–21] adressiert werden. Dies umfasst vor allem die Anforderungen an digital unterstützte Strukturen und Prozesse der Versorgung im Kontext von Gesundheit und Pflege – auch mit Blick auf den sicheren Umgang mit IT-Lösungen. Zurückführen lässt sich diese Schwerpunktsetzung unter anderem auf die Ausrichtung der Studiengänge in den Bereichen des Managements und der Versorgung. Insbesondere in den thematisch eher breiter ausgelegten bzw. stärker PH-orientierten Studiengängen sowie bei Modulen unter Berücksichtigung von Querschnittsbereichen lassen sich Schwerpunkte bezüglich der Bedeutung der Digitalisierung auf die Determinanten von Gesundheit und Krankheit (inkl. einer Reflexion der ethischen Implikation), auf die Gesundheitskommunikation sowie im Kontext der Forschung identifizieren. Unter Berücksichtigung der von der Weltgesundheitsorganisation (WHO) vorgeschlagenen EPHO [43] scheinen jedoch, soweit die begrenzte Informationstiefe der Modulhandbücher eine entsprechende Bewertung zulässt, die Bereiche der Surveillance, Krisenplanung und -reaktion sowie Governance (im Sinne von Führung und Verantwortung bei gesundheitsbezogenen Fragen) noch unzureichend adressiert zu werden.

Um der multidisziplinären, praxisorientierten und berufsqualifizierenden Ausrichtung von PH-orientierten Studiengängen als Grundsatz der Qualifizierung gerecht zu werden, gilt es, dem Transfer der Forschung in die Praxis durch die Studiengänge Rechnung zu tragen und die Studierenden zu befähigen, auf der Basis von wissenschaftlichem Know-how die gesundheitsbezogene Praxis zu gestalten und zu verändern [44]. Um Veränderungen durch die digitale Transformation mitzugestalten, ist es notwendig, relevante DiPH-Bezüge in PH-orientierten Studiengängen zu berücksichtigen. Das Fehlen solcher Lehrinhalte könnte dazu führen, dass zukünftige Absolvent*innen im Bereich PH nicht optimal auf die Anforderungen einer digitalisierten Gesundheitslandschaft vorbereitet werden.

Demnach sollten Überlegungen angestellt werden, ob und wie ein grundlegender Leitfaden an Kompetenzen zu DiPH in allen einschlägigen Curricula von PH-orientierten Studiengängen verankert werden kann. Ein gemeinsamer Kanon von Kompetenzen kann Orientierung bei der Gestaltung und Abstimmung von Ausbildungsangeboten bieten und die Aufgaben von PH in der öffentlichen Wahrnehmung schärfen. Dies sollte in engem Austausch mit den Fachgesellschaften sowie mit europäischen Initiativen, wie der ASPHER, erfolgen [26]. Ein Augenmerk sollte hierbei auf die Heterogenität von Denominationen der PH-orientierten Studiengänge sowie der DiPH-Schwerpunkte gelegt werden. Zugleich ist anzumerken, dass relevante Kompetenzen für PH-Fachkräfte auch in Studiengängen außerhalb der als PH-orientiert identifizierten Studiengänge erworben werden können.

### Stärken und Limitationen

Durch das umfassende Screening von deutschen Studiengängen im Bereich Gesundheit konnte die PH-Orientierung in Studiengängen identifiziert und in der Analyse berücksichtigt werden, auch wenn der Begriff PH nicht unmittelbar im Namen des Studiengangs enthalten war. Somit bietet die MHBA eine umfassende Analyse der DiPH-bezogenen Module in PH-orientierten Studiengängen. Limitiert wird die MHBA dadurch, dass Studiengänge, zu denen kein Modulhandbuch identifiziert werden konnte, nicht berücksichtigt wurden. Auch lag dem Screeningprozess ein enges Verständnis von PH zugrunde, weshalb die zugrunde liegenden Kriterien zum Einschluss von Studiengängen eng gefasst waren und relevante Studiengänge womöglich nicht berücksichtigt wurden. Dies könnte zu einer unvollständigen Darstellung der DiPH-bezogenen Module geführt haben und beeinflusst somit den Anspruch auf Vollständigkeit. Zum anderen wird die MHBA durch die Abbildung einer Momentaufnahme des Lehrsolls limitiert. Abweichungen in der tatsächlichen Lehre durch flexible Lehranpassungen durch Dozent*innen sowie durch vorgenommene Änderungen in den MHB nach der abgeschlossenen MHBA sind nicht auszuschließen. Das Screening der Studiengänge wurde von mehreren Personen durchgeführt, wobei die deskriptive Inhaltsanalyse der Studiengänge stets von Autor*innen erfolgte, die selbst nicht den analysierten Studiengängen angehören. Der Kodierungs- und Analyseprozess erwies sich insgesamt als herausfordernd, da die Modulhandbücher keine einheitliche formale und inhaltliche Struktur aufwiesen.

## Fazit

DiPH-bezogene Module sind an verhältnismäßig wenigen PH-orientierten Studiengängen verortet. Die Inhalte der identifizierten DiPH-bezogenen Module sind dabei heterogen, da die einzelnen Standorte ihren Studienangeboten eine spezifische Ausrichtung geben. Dennoch decken sie sich mit dem bestehenden Wissen über den Gegenstand, der Terminologie, den Anwendungsfeldern und Methoden von DiPH. Es ist sowohl zu erwarten als auch erstrebenswert, dass sich der spezifische Lehr- und Lerngegenstand DiPH in der PH-Lehre weiterentwickelt und diskutiert wird, um aktuelle Entwicklungen in PH-Professionen sowie den Forschungsbedarf in diesen Disziplinen zu berücksichtigen und die Qualität der Studiengänge zu gewährleisten. Eine Diskussion und Analyse der bisherigen Ergebnisse mit Lehrenden und Studierenden der PH-orientierten Studiengängen wären relevant, um zu verstehen, wie die aufgezeigten Inhalte der Modulhandbücher in den jeweiligen Studiengängen interpretiert und konzeptualisiert werden. Auch würde eine Befragung Aufschluss über die Relevanz von DiPH-bezogenen Modulen in PH-orientierten Studiengängen geben, die nach dem Stand der MHBA keine DiPH-bezogenen Module anbieten.

Ebenso wäre die Befragung von Absolvent*innen im Bereich PH mit anschließender Berufspraxis im Bereich DiPH aufschlussreich, um Wissen bezüglich ihrer Einschätzung zur Vorbereitung auf die Berufspraxis zu generieren und weitere Anforderungen an die akademische Ausbildung ableiten zu können. Grundsätzlich sollten Studierende explizit einbezogen werden, um ihr Wissen über aktuelle Anforderungen einzubringen. Die Potenziale einer Neuausrichtung der PH-Ausbildung im Sinne einer Erweiterung um DiPH-bezogene Inhalte liegen in ihrem beruflichen Selbstverständnis. Dieses reagiert auf neue Bedarfe sowie Bedürfnisse und füllt Lücken im System, die durch aktuelle Entwicklungen entstehen. Ferner gilt es zu diskutieren, inwieweit Empfehlungen für ein gemeinsames Kerncurriculum im Bereich DiPH dazu beitragen können, auch zukünftig die Qualität des Studienangebots im Bereich PH in Deutschland zu sichern und die Erwartungen der Zielgruppen (in der Studiengangwahl, der Gesundheitswirtschaft und der Forschung) zu erfüllen. Eine solche Überlegung könnte dazu beitragen, eine kohärente und einheitliche Grundlage für die akademische Ausbildung zukünftiger PH-Fachkräfte im Bereich DiPH zu schaffen, die realweltliche Anforderungen an die akademische Berufspraxis im Bereich DiPH berücksichtigt.

### Supplementary Information




