# Supplementary material for: How much digital public health is in public health degree programs? A systematic analysis of module handbooks in German full-time study programs at public colleges and universities
Source: Bundesgesundheitsblatt Gesundheitsforschung Gesundheitsschutz. 2024 Mar 4;67(3):339–50. [Article in German] doi: 10.1007/s00103-024-03844-2 (PMC10927875; doi:10.1007/s00103-024-03844-2)
Supplement: Supplementary file 1 [file 103_2024_3844_MOESM1_ESM.pdf]

Onlinematerial

Beitrag von Albrecht et al. (Bundesgesundheitsblatt 3/2024)

Tabelle Z1: Kategoriensystem zur Inhaltsanalyse der Modulhandbücher

**Kategoriensystem**
Public Health / Sozialmedizin<br>• Public Health Profession<br>• Gesellschaftliche Transformation
Public Health-Forschungsmethoden / Sozial- und Versorgungsforschung<br>• Entwicklung und Evaluation<br>• Akzeptanzforschung<br>• Technikfolgenabschätzungen (iwS)<br>• Nutzerorientierung
Epidemiologie
Gesundheitsförderung / Prävention / Gesundheitserziehung<br>• Digitale Kompetenzen<br>• Gesundheitskompetenzen
Gesundheits- und Krankheitsdeterminanten / soziale Ungleichheit
Gesundheitsökonomie und -management<br>• Finanzierung
Gesundheitspolitik und -system<br>• eHealth / Gesundheitstelematik / Telemedizin<br>• Veränderungen Gesundheitssystem / -versorgung
Querschnitt: IT und Technik
Querschnitt: Gesundheitskommunikation
Querschnitt: Ethik und Recht

Tabelle Z2: Übersicht der Public Health-bezogenen Studiengänge mit digital Public Health-Bezug (N=16)

ID	Hochschule / Universität	Denomination des Studiengangs	Veröffentlichungs-datum des Modulhandbuchs	Bachelor / Master	Gesamt Credit Points für Digital Public Health
A	Hochschule Aalen - Technik und Wirtschaft	Gesundheitsmanagement	23.02.2021	Bachelor	25
B	Alice Salomon Hochschule Berlin	Management und Versorgung im Gesundheitswesen	25.05.2020	Bachelor	5
C	Universität Bielefeld	Health Communication	22.02.2023	Bachelor	35
D	Hochschule für Gesundheit Bochum - University of Applied Sciences	Gesundheitsdaten und Digitalisierung	26.05.2021	Bachelor	118
E		Pflege	28.11.2019	Bachelor	32
F	Technische Universität Dresden	Gesundheitswissenschaften/Public Health	Ohne Datum	Master	5
G	Hochschule Fulda - University of Applied Sciences	Management in der Gesundheitsversorgung	19.01.2022	Bachelor	30
H		Interprofessionelles Management in der Gesundheitsversorgung	29.11.2021	Master	5
I	Technische Hochschule Mittelhessen - THM Gießen	Public Health	04.12.2019	Master	24
J	Hochschule für Angewandte Wissenschaften Hamburg	Gesundheitswissenschaften	16.12.2021	Bachelor	10
K		Health Sciences	18.11.2021	Master	17
L	Hochschule für angewandte Wissenschaften Kempten	Gesundheitswirtschaft	20.01.2021	Bachelor	21
M	Hochschule Niederrhein	Health Care	23.09.2020	Master	6
N	Universität Siegen	Digital Biomedical and Health Sciences (Vertiefung Digital Public Health)	17.03.2022	Bachelor	117
O		Digital Public Health	24.06.2022	Master	75
P	Hochschule Furtwangen	Angewandte Gesundheitswissenschaften	27.01.2021	Bachelor	66
